# Revisiting the Genetics of Hypertrophic Cardiomyopathy: From Sarcomeres to Polygenic Modulation and Clinical Translation

**DOI:** 10.3390/jcm15062327

**Published:** 2026-03-18

**Authors:** Maria Cristina Carella, Marco Maria Dicorato, Paolo Basile, Ilaria Dentamaro, Daniela Santoro, Eugenio Carulli, Michele Davide Latorre, Eduardo Urgesi, Francesco Monitillo, Nicoletta Resta, Gianluca Pontone, Marco Matteo Ciccone, Andrea Igoren Guaricci, Cinzia Forleo

**Affiliations:** 1 University Cardiology Unit, Interdisciplinary Department of Medicine, Polyclinic University Hospital, University of Bari “Aldo Moro”, 70124 Bari, Italy; m.dicorato20@phd.uniba.it (M.M.D.); paolo.basile@uniba.it (P.B.); ilaria.dentamaro@hotmail.it (I.D.); danina2003@libero.it (D.S.); cinzia.forleo@uniba.it (C.F.); 2 Medical Genetics Unit, Department of Precision and Regenerative Medicine and Ionian Area (DiMePRe-J), University of Bari “Aldo Moro”, University Hospital Consortium, Polyclinic of Bari, 70124 Bari, Italy; 3 Department of Perioperative Cardiology and Cardiovascular Imaging, IRCCS Centro Cardiologico Monzino, 20138 Milan, Italy; 4 Department of Biomedical, Surgical and Dental Sciences, University of Milan, 20100 Milan, Italy

**Keywords:** hypertrophic cardiomyopathy, cardiogenetics, sarcomeric genes, *MYBPC3*, *MYH7*, incomplete penetrance, polygenic risk score, genetic modifiers, deep intronic variants, phenocopies, precision cardiology

## Abstract

Hypertrophic cardiomyopathy (HCM), the most common inherited cardiomyopathy, represents a paradigmatic condition for precision cardiovascular medicine. Once regarded as a monogenic autosomal dominant disorder driven by rare sarcomeric variants, HCM is now recognized as a genetically complex disease characterized by incomplete penetrance, variable expressivity, and heterogeneous clinical trajectories. This review summarizes current evidence on the evolving genetic architecture of HCM, emphasizing the predominant role of definitively validated sarcomeric genes, particularly *MYBPC3* and *MYH7*, and the clinical value of gene panel expansion. Phenotypic variability reflects interactions among variant classes, gene-specific mechanisms, and modifying factors. Differences between missense and truncating variants, haploinsufficiency and poison-peptide effects, allelic imbalance, and age-dependent penetrance contribute to diverse disease expression. Emerging data further support oligogenic inheritance and polygenic modulation, with genome-wide association studies and polygenic risk scores elucidating their contribution to disease susceptibility and variability, especially in genotype-negative patients and carriers of rare variants. We also address genes with emerging evidence and underrecognized pathogenic mechanisms, including deep intronic and splice-altering variants that may explain part of the missing heritability. The importance of distinguishing phenocopies is highlighted, advocating for phenotype-anchored diagnostic pathways integrating clinical assessment, multimodality imaging, and targeted genetic testing. Overall, contemporary data support a targeted, gene-validity-driven approach to genetic testing, where molecular findings primarily inform diagnosis and cascade screening, while risk stratification remains phenotype-led and longitudinal. Future progress will depend on integrative models combining rare variants, polygenic background, imaging, and biomarkers to translate genetic complexity into actionable precision care.

## 1. Introduction

Hypertrophic cardiomyopathy (HCM) is the most common inherited cardiomyopathy, with an estimated prevalence ranging from 1:200 to 1:500 in the general population [1,2,3]. It is characterized predominantly by unexplained left ventricular hypertrophy, myocyte disarray, and interstitial fibrosis and represents a major cause of heart failure, atrial fibrillation (AF), and sudden cardiac death (SCD), particularly in young individuals and athletes [4,5,6]. Since the first identification of pathogenic variants in sarcomeric genes more than three decades ago [7,8,9], HCM has become a prototype disease for the application of genetics in cardiovascular medicine.

**Figure 1:**
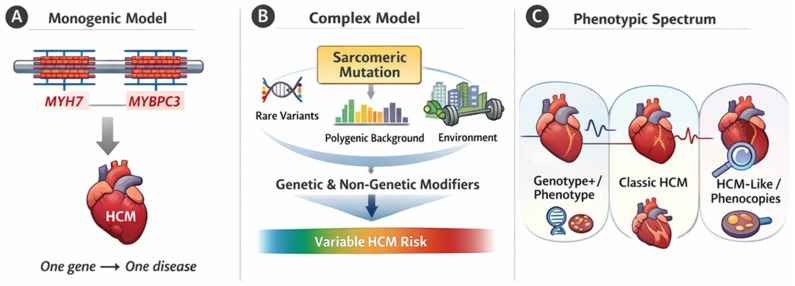
From monogenic disease to near-Mendelian and polygenic hypertrophic cardiomyopathy. Panel (**A**) illustrates the traditional monogenic model of HCM, in which a single pathogenic variant in a sarcomeric gene (most commonly *MYH7* or *MYBPC3*) is considered sufficient to cause disease (“one gene–one disease”). Panel (**B**) depicts the contemporary, more complex model, whereby a rare sarcomeric pathogenic variant represents the primary genetic substrate but disease penetrance and expressivity are modulated by additional factors, including other rare variants, the polygenic background, and environmental or clinical modifiers, resulting in a continuum of HCM risk. Panel (**C**) summarizes the resulting phenotypic spectrum, ranging from genotype-positive/phenotype-negative individuals, through classic sarcomeric HCM, to HCM-like phenotypes and phenocopies driven by distinct molecular mechanisms. This framework highlights that genotype alone is insufficient to fully explain disease expression and supports an integrated, phenotype-anchored approach to clinical interpretation.

Historically, HCM has been considered a classic autosomal dominant monogenic disorder caused mainly by pathogenic variants in genes encoding sarcomeric proteins, such as MYH7 and MYBPC3. However, clinical experience and large cohort studies have consistently demonstrated marked inter- and intrafamilial variability, incomplete and age-dependent penetrance, and heterogeneous clinical outcomes, even among carriers of the same genetic variant [10,11,12]. These observations have challenged the traditional one-gene–one-disease paradigm and prompted a more nuanced view of HCM as a condition influenced by multiple genetic and non-genetic factors [13,14,15] (Figure 1).

Recent position papers from international societies and the European Society of Cardiology (ESC) have highlighted several paradigm shifts in cardiovascular genetics that are particularly relevant to HCM [16]. These include: (i) the critical importance of gene–disease validity when designing and interpreting genetic tests; (ii) the recognition that expanding gene panels indiscriminately increases the burden of variants of uncertain significance (VUS) without substantially improving diagnostic yield; and (iii) the growing appreciation of oligogenic inheritance and polygenic background as modulators of disease expression. In parallel, there is increasing emphasis on the active role of the clinical cardiologist in coordinating the entire genetic pathway—from phenotypic characterization and test selection to result disclosure, cascade screening, and longitudinal management of patients and relatives [17,18,19].

Over the last 20 years, the rapid evolution of molecular diagnostic techniques—most notably next-generation sequencing (NGS)—has profoundly reshaped the clinical approach to HCM and other inherited cardiovascular conditions [17,20,21]. Genetic testing is no longer confined to research settings or highly specialized centers but has progressively entered routine clinical practice, as emphasized by recent international consensus documents and guidelines [16,18]. These documents underscore that cardiologists should be familiar not only with the indications for genetic testing, but also with its limitations, interpretation, and downstream clinical consequences. We seek to clarify what clinicians should test, how results should be interpreted, and how genetic findings can meaningfully inform patient care and family management in the era of precision cardiology.

### Methods

This article was conceived as a narrative review aimed at summarizing the evolving genetic architecture of hypertrophic cardiomyopathy and its implications for clinical practice. A literature search was primarily conducted using the PubMed/MEDLINE database to identify relevant publications on HCM genetics, sarcomeric and non-sarcomeric genes, genetic modifiers, polygenic risk, and phenocopies, focusing primarily on articles published within the last ten years. Searches included combinations of keywords such as “hypertrophic cardiomyopathy”, “cardiogenetics”, “sarcomeric genes”, “MYBPC3”, “MYH7”, “polygenic risk score”, “genetic modifiers”, and “deep intronic variants”.

Articles published in English were considered, with particular emphasis on recent studies, large cohort analyses, genome-wide association studies, systematic reviews, and international guideline or consensus documents relevant to cardiomyopathy genetics. Reference lists of key publications were also screened to identify additional relevant sources.

## 2. Sarcomeric Genes as the Cornerstone of Hypertrophic Cardiomyopathy Genetics

**Figure 2:**
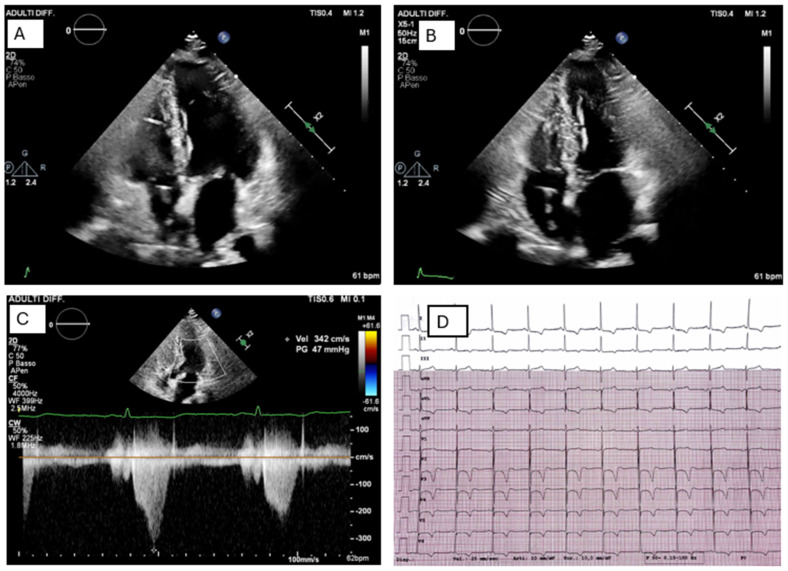
Sarcomeric HCM in a genetically characterized patient. Comprehensive molecular analysis of an 80-gene cardiomyopathy panel identified a pathogenic heterozygous variant in TNNI3 and a heterozygous VUS in CAV3. Apical four-chamber echocardiographic views obtained in diastole (Panel (**A**)) and systole (Panel (**B**)) demonstrate moderate asymmetric septal and apical hypertrophy (interventricular septum 15 mm, apical thickness 13 mm, posterior wall 10 mm), with preserved global systolic function (LVEF 56%). Panel (**C**) shows a dynamic left ventricular outflow tract (LVOT) gradient, increasing from 15 mmHg at rest to 47 mmHg during Valsalva maneuver, with the characteristic dagger-shaped Doppler profile. Panel (**D**) displays a 12-lead ECG revealing an atrial paced rhythm from a pacemaker programmed in AAI mode at 60 bpm, along with electrocardiographic signs of left ventricular hypertrophy with strain pattern.

HCM displays substantial genetic heterogeneity, but its inherited architecture is dominated by rare variation in a limited set of sarcomeric genes, a finding that has been consistently supported by large case–control analyses and registry data [22,23]. In contemporary clinical practice, a pathogenic/likely pathogenic (P/LP) variant is identifiable in ~30% of unselected “all-comers” with HCM, whereas the diagnostic yield rises to >60% in patients with a family history, underscoring how pre-test probability (familial disease, early onset) strongly modulates the likelihood of a molecular diagnosis [18,24]. At the gene level, sarcomeric HCM is largely attributable to thick filament genes (MYBPC3, MYH7, MYL2, and MYL3), with MYBPC3 accounting for ~40–50% of genotype-positive cases and MYH7 for ~33%, while thin filament variants (TNNT2, TNNI3, TPM1, ACTC1) are comparatively rarer (Figure 2); for instance, TNNT2 variants are reported in roughly ~2–5% of cases in clinical series, highlighting the skewed distribution of genetic causality across sarcomeric pathways. Importantly, although many genes have been historically “implicated” in HCM, multiple lines of evidence indicate that variants outside the sarcomere are much less frequent and often less securely established as disease-causal, reinforcing the need to prioritize robust gene–disease validity when designing and interpreting genetic panels [25].

From a clinical standpoint, it is also critical to distinguish gene validity (is the gene truly associated with HCM?) from variant pathogenicity (is this specific variant disease-causing?) and from clinical meaning (what does this imply for penetrance, prognosis, and family management). The ClinGen reappraisal emphasizes that gene-level evidence is dynamic: in the 2024 update, multiple genes changed classification, with several downgraded to disputed (discouraging diagnostic reporting), while some were upgraded based on new data—illustrating why indiscriminate panel expansion can inflate uncertainty rather than improve actionable yield [26].

The SHaRe registry provides particularly strong evidence that sarcomeric status is not a purely “descriptive” label but carries prognostic signal: patients with P/LP sarcomere variants (SARC+) had an approximately >2-fold higher risk of adverse outcomes compared with SARC− patients, with the strongest relative hazard observed for ventricular arrhythmias (reported HR ~2.8 in multivariable models), whereas carriers of sarcomeric VUS showed an intermediate risk profile—supporting the concept of a graded genetic contribution to lifetime disease burden [27]. SHaRe also highlights that the temporal pattern of morbidity is genotype-influenced: ventricular arrhythmias cluster more in those diagnosed at younger ages (with much higher event proportions < 40 years) while HF and AF emerge as dominant components of lifetime burden later on, reinforcing the need for lifelong surveillance even in patients diagnosed young and initially stable [28].

Collectively, these findings support a contemporary definition of sarcomeric HCM as a condition in which rare pathogenic variants in sarcomeric genes represent the principal monogenic substrate and are associated with a higher-risk disease course while being insufficient to fully account for incomplete penetrance, variable expressivity, and inter-individual heterogeneity in clinical trajectories. This framework provides the rationale for addressing phenocopies and diagnostic pitfalls, as well as additional genetic modifiers and complex inheritance models that refine risk beyond the binary presence of a single rare variant.

### Beyond the Sarcomere: Gene–Disease Validity and Diagnostic Pitfalls in Hypertrophic Cardiomyopathy

The progressive expansion of NGS has profoundly increased the accessibility of genetic testing in HCM [20,29]; however, it has also exposed a critical distinction between technical feasibility and clinical interpretability [30]. Although more than 50 genes have been historically reported in association with HCM, converging evidence from large case–control studies and systematic curation initiatives indicates that a restricted subset of genes accounts for the vast majority of genetically confirmed cases [22,31]. Within this restricted subset, genes encoding sarcomeric proteins represent the strongest and most consistently validated contributors to HCM. According to contemporary gene–disease validity frameworks, MYBPC3 and MYH7 have definitive evidence for causality, together accounting for the largest proportion of genotype-positive cases [22,26]. Additional sarcomeric genes with strong or definitive evidence include TNNT2, TNNI3, TPM1, MYL2, MYL3, and ACTC1, whereas other genes historically labelled as “HCM-associated” show only limited or disputed evidence when evaluated using rigorous statistical and experimental criteria [32].

Importantly, large-scale reappraisal has demonstrated that many non-sarcomeric genes initially implicated in HCM—often through candidate gene approaches, small pedigrees, or biological plausibility—do not show significant enrichment of rare pathogenic variants in HCM cohorts compared with population controls [22,31]. The landmark case–control analysis by Walsh and colleagues showed that inclusion of such genes in diagnostic panels does not meaningfully increase diagnostic yield, but instead results in a disproportionate rise in VUS, thereby increasing interpretative ambiguity without clear clinical benefit. This issue has become increasingly relevant with the expansion of NGS panels, which allow the simultaneous interrogation of a large number of genes but inevitably increase the likelihood of detecting VUS. The interpretation of VUS represents a major challenge in clinical practice, as these variants cannot be used to guide diagnosis, cascade screening, or clinical management without additional evidence. Consequently, careful variant interpretation following established classification frameworks, together with segregation analysis, functional studies, and periodic re-evaluation of variant pathogenicity, is essential to minimize misinterpretation and ensure appropriate use of genetic information in the clinical setting.

**Figure 3:**
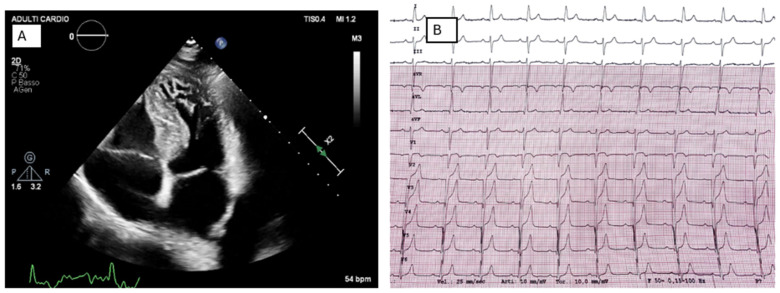
Non-obstructive HCM in a genetically negative patient. The patient underwent serial genetic testing over time using progressively expanded panels. A comprehensive molecular analysis of 174 cardiomyopathy-associated genes identified a heterozygous VUS in LAMA4. Expansion of the genetic panel did not result in any change in diagnosis or clinical management. Panel (**A**). Transthoracic echocardiography shows a normal-sized left ventricle with severe asymmetric septal hypertrophy (anterior IVS 20 mm; posterior IVS 18 mm), preserved global systolic function (biplane Simpson LVEF 63%), and no regional wall motion abnormalities. Apical hypertrabeculation is noted. Panel (**B**). Twelve-lead ECG demonstrates sinus rhythm at 62 bpm, first-degree atrioventricular block (PR interval 220 ms), left anterior fascicular block, and electrocardiographic signs of left ventricular hypertrophy. Poor R-wave progression from V1 to V4 is observed.

Failure to account for gene–disease validity represents a major diagnostic pitfall. Reporting variants in genes with limited or disputed evidence may lead to inappropriate attribution of causality, unnecessary surveillance of relatives, or false reassurance when variants are misclassified as explanatory [33] (Figure 3). Conversely, restricting genetic testing to genes with strong or definitive evidence improves the signal-to-noise ratio, enhances the proportion of actionable results, and facilitates more reliable cascade screening and counselling. Crucially, gene–disease validity must be distinguished from variant-level pathogenicity and from clinical expressivity. Even within the restricted subset of definitively associated genes, individual variants display incomplete penetrance and variable expressivity, reinforcing the need for integrated interpretation that combines genetic findings with clinical phenotype, imaging, and longitudinal follow-up.

## 3. Incomplete Penetrance and Variable Expressivity: Why Genotype Is Not Destiny in HCM

Incomplete penetrance and variable expressivity are defining properties of HCM and represent a central reason why a P/LP variant often has limited standalone prognostic utility. Even within families sharing the same causal variant, clinical expression may range from a completely normal phenotype to severe hypertrophy, HF, AF, or malignant ventricular arrhythmias, underscoring that “Mendelian” does not necessarily mean deterministic at the individual level [16,18,31].

The most rigorous quantification of penetrance in genetic HCM has recently been provided by a recent meta-analysis, which makes explicit that penetrance is context-dependent and strongly influenced by how variant carriers are ascertained [34]. In family studies (cascade screening of relatives carrying the known familial P/LP variant), pooled cross-sectional penetrance across sarcomere or sarcomere-related genes was ~57%, with substantial heterogeneity by gene (e.g., ~55% for MYBPC3, ~64–65% for MYH7, ~60–62% for TNNT2/TNNI3, and lower estimates for some genes such as MYL3). By contrast, in genotype-first population studies where P/LP variants are found incidentally in the general population, penetrance of “conventional” HCM was markedly lower (~11% overall), illustrating that many carriers may not reach the classical diagnostic threshold even by midlife. Longitudinal data further refine this picture by separating prevalent penetrance from phenotypic conversion. In longitudinal family-based cohorts of genotype-positive/phenotype-negative relatives, phenotypic conversion to overt left ventricle hypertrophy (LVH) occurred in roughly ~15% over ~8 years of follow-up (starting from a mean baseline age in adolescence), again with gene-dependent variability (e.g., higher short-term conversion for MYH7 than MYBPC3). These estimates highlight why a negative phenotype at one time point does not equal “non-penetrant forever,” and why the appropriate clinical construct is age-dependent risk rather than a binary penetrant/non-penetrant label.

Beyond the presence or absence of LVH, increasing evidence indicates that sarcomere variant carriers may exhibit a spectrum of subclinical traits—diastolic dysfunction, energetic abnormalities, fibrosis by cardiac magnetic resonance (CMR), myocardial crypts, mitral valve and microstructural abnormalities, and microvascular dysfunction—sometimes preceding overt hypertrophy and potentially contributing to symptoms or risk [31,35,36]. This broader phenotypic continuum challenges the classical “threshold-based” definition of penetrance and supports the notion that penetrance should be conceptualized not only as LVH expression but also as the emergence of measurable intermediate phenotypes that may carry clinical relevance [1,37].

Finally, to link variant class to clinical variability, it is useful to distinguish missense mutations, typically observed in *MYH7*, *TNNT2*, *TNNI3*, and *TPM1*, from truncating mutations, which are classically associated with *MYBPC3* [38,39].

Missense variants often generate a relatively stable mutant protein that can be incorporated into the sarcomere (the so-called “poison peptide” mechanism or dominant-negative mechanism). In this context, the mutant protein co-assembles with the wild-type protein within the contractile apparatus and directly interferes with normal sarcomere function, thereby altering cross-bridge kinetics, increasing myofilament Ca^2+^ sensitivity, and raising the energetic cost of contraction [40,41,42]. Because these mutant proteins actively perturb sarcomeric function, the functional impact of these variants depends critically on the affected domain and protein, thereby accounting for marked intra-gene and intra-familial heterogeneity.

In contrast, many *MYBPC3* truncating variants act predominantly through haploinsufficiency, a mechanism in which one allele fails to produce a functional protein and the remaining wild-type allele is insufficient to maintain normal protein levels. As a result, reduced availability of cardiac myosin-binding protein C (cMyBP-C) impairs sarcomere stability and contractile regulation rather than producing a toxic truncated protein.

In this dose-threshold model, contractile dysfunction emerges when cMyBP-C levels fall below a critical threshold (reported at approximately ~73% in experimental models), consistent with a pattern of often delayed yet substantial lifetime penetrance [43,44]. This framework integrates with the concept of a “toxic dose”, whereby in heterozygous carriers the relative proportion of mutant versus wild-type protein may vary among cardiomyocytes due to allelic imbalance and age-dependent decline in protein quality control systems, thereby contributing to incomplete penetrance and non-uniform phenotypic conversion over time [44,45,46]. Consistent with these mechanisms, disease penetrance and age of clinical expression may differ across sarcomeric genes: variants in *MYH7* are frequently associated with earlier phenotypic manifestation and relatively higher penetrance, whereas *MYBPC3* truncating variants often display age-dependent penetrance and later clinical onset [34,39,40]. From a clinical perspective, these variant-specific mechanisms help explain part of the variability in disease expression observed among patients and families with HCM.

The biological basis of variable expressivity is multifactorial and includes (i) allelic heterogeneity (many variants are “private” to single families), (ii) variant class effects (e.g., differences between truncating and missense variants, gene-specific mechanisms), and (iii) the contribution of additional genetic and non-genetic modifiers [31,47]. Founder effects (particularly for some MYBPC3 variants) illustrate how identical variants can show divergent clinical trajectories across populations and families, consistent with modulatory influences beyond the primary variant [48,49].

A key concept in contemporary cardiogenetics is that the penetrance and expressivity of Mendelian variants are shaped by additional genetic modifiers—including rare intermediate-effect variants and common variants captured by polygenic risk scores—as well as environmental and clinical exposures [16,17,18,50,51]. This “near-Mendelian” framework helps explain why carriers of the same P/LP sarcomeric variant may follow markedly different clinical trajectories.

Clinically, these observations argue against an overly simplistic genotype-based prognostic approach and instead support an integrated framework in which genetic findings primarily inform diagnosis and cascade screening, while individual risk stratification remains largely phenotype-driven and longitudinal. At the same time, appreciating the context-dependent penetrance of P variants has practical implications for clinical management: relatives identified through cascade screening who carry P/LP variants require structured surveillance, with timing and intensity tailored to age, family history, and emerging phenotype. Importantly, large cohort studies have shown that sarcomeric genotype is nevertheless associated with a higher lifetime burden of disease, with carriers of pathogenic variants demonstrating earlier disease onset and an increased risk of adverse outcomes, including ventricular arrhythmias and heart failure, compared with genotype-negative patients [27,34,51]. Looking forward, emerging genetic insights may progressively refine risk assessment strategies in HCM. Beyond the presence of a single rare variant, factors such as variant class, gene-specific mechanisms, oligogenic inheritance, and polygenic background are increasingly recognized as contributors to interindividual variability in disease expression and clinical outcomes. The integration of rare pathogenic variants with polygenic risk scores and other genetic modifiers, together with clinical phenotype, imaging parameters, and circulating biomarkers, may enable the development of multidimensional predictive models. Such integrative approaches have the potential to support more personalized risk stratification and therapeutic decision-making in HCM, potentially informing the timing and intensity of clinical surveillance, the selection of candidates for implantable cardioverter-defibrillator implantation for primary prevention, and the initiation of emerging disease-modifying therapies, including targeted pharmacological treatments such as cardiac myosin inhibitors and gene therapies, in patients most likely to benefit from early intervention. According to current clinical guidelines, only a limited number of genes—including *LMNA*, *TMEM43*, and *PLN*, as well as truncating variants of *FLNC*, *DSP*, and *RBM20*—are currently considered to have direct implications for therapeutic decision-making in patients with dilated cardiomyopathy (DCM) or non-dilated left ventricular cardiomyopathy, but not in patients with HCM.

## 4. Genetic Modifiers and Polygenic Background

**Figure 4:**
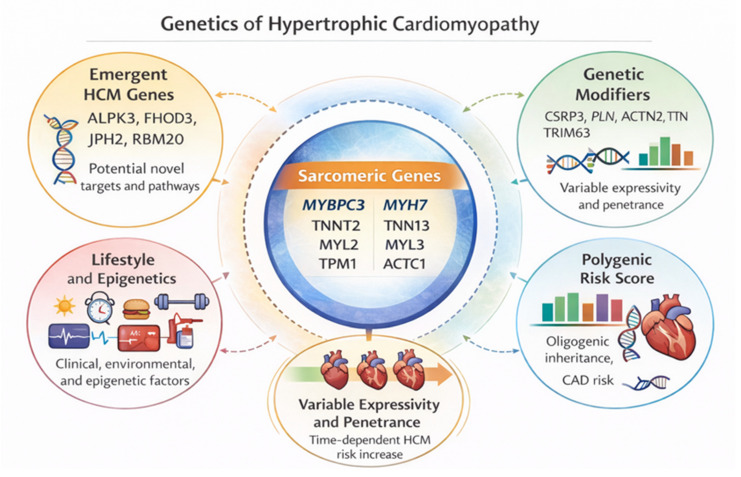
Multilayered genetic architecture of hypertrophic cardiomyopathy. Sarcomeric genes with definitive or strong gene–disease validity (*MYBPC3*, *MYH7*, *TNNT2*, *TNNI3*, *TPM1*, *MYL2*, *MYL3*, *ACTC1*) represent the core monogenic substrate of HCM. Surrounding this central axis, multiple additional layers modulate disease penetrance and expressivity. These include emerging HCM-associated genes (e.g., *ALPK3*, *FHOD3*, *JPH2*), rare and intermediate-effect genetic modifiers, and the polygenic background captured by genetic risk scores, which collectively influence disease susceptibility and phenotypic severity. Non-genetic factors, including lifestyle, environmental exposures, and epigenetic mechanisms, further contribute to interindividual variability. The interaction among these components results in age-dependent penetrance and a broad spectrum of clinical expressivity, supporting a near-Mendelian and oligogenic model of HCM rather than a strictly deterministic monogenic disease.

A growing body of evidence supports the concept that HCM, even when initiated by a rare pathogenic sarcomeric variant, frequently follows a “near-Mendelian” model in which penetrance and expressivity are shaped by additional genetic layers, including intermediate-effect variants and a polygenic background, together with non-genetic exposures [2,13,51,52] (Figure 4). This framework explicitly distinguishes large-effect Mendelian variants from common variants of small effect that can collectively modulate disease severity, and it provides a mechanistic explanation for the striking within-family heterogeneity observed in sarcomeric HCM [34,53,54].

Genome-wide association studies (GWAS) and genetic risk scores show that common DNA variation contributes measurably to HCM susceptibility, particularly among genotype-negative cases [55,56]. In previous GWAS, heritability estimates derived from common variants were substantially higher in sarcomere-negative HCM than in sarcomere-positive disease (reported ~34% vs. ~16%), indicating a stronger polygenic contribution when a monogenic driver is not identified [57]. In the same line, a genetic risk score built from HCM-associated single nucleotide polymorphisms (SNPs) stratified risk across the population, with individuals in the highest risk quintile exhibiting more than a twofold higher odds of HCM compared to the reference distribution. This evidence supports the idea that common variants can be collectively relevant for disease liability [10,58].

The polygenic background is not only relevant to disease “susceptibility” but also to phenotypic expressivity among carriers of rare variants. Polygenic risk scores (PRS) can explain a portion of phenotypic variability in individuals who already carry rare pathogenic variants, implying that the polygenic load can act as a quantitative modifier, shifting an individual toward earlier onset, greater hypertrophy, or a more symptomatic course, rather than behaving as an on/off determinant [13,16,54]. This helps reconcile why a clearly pathogenic variant may have high diagnostic value but limited standalone prognostic precision. Indeed, an additional and clinically intriguing dimension is that the common-variant genetic architecture appears to overlap across cardiomyopathy phenotypes, often with opposite directions of effect for HCM versus DCM [59,60,61]. Recent summaries of the GWAS literature have identified shared loci and pathways influencing left ventricular structure and function in the general population, whereby alleles predisposing to greater wall thickness and enhanced contractility tend to align with HCM susceptibility while being protective against DCM, and conversely, variants favouring ventricular dilation and systolic impairment increase DCM risk while mitigating hypertrophic remodelling. Within this framework, loci such as *HSPB7*, *BAG3*, and LMNA-related mechanotransduction pathways exemplify a bidirectional genetic architecture, acting not as primary disease drivers but as phenotypic modifiers that bias myocardial adaptation toward hypertrophy or dilation depending on the host genetic and biological context [13,57,58,62]. This bidirectional genetic correlation suggests that part of the phenotypic spectrum across cardiomyopathies may reflect a shared biological substrate modulated by variant directionality and background genetic context.

Despite these advances, the current clinical applicability of PRS in HCM remains limited. Most PRS have been derived from research cohorts and require further validation across diverse populations before routine clinical implementation. In addition, the thresholds for clinical decision-making and their integration with established risk markers, such as imaging findings, family history, and clinical phenotype, remain uncertain. At present, therefore, polygenic scores should be considered primarily a research tool that may contribute to future integrative risk prediction models rather than a component of routine clinical risk stratification.

## 5. Emerging Genes in Hypertrophic Cardiomyopathy

In recent years, the genetic architecture of HCM has expanded beyond the core sarcomeric genes, with the emergence of non-sarcomeric (or sarcomere-associated) genes showing reproducible associations with HCM-like phenotypes [20,29] (Table 1).

Among these, *JPH2* (junctophilin-2) is involved in excitation–contraction coupling homeostasis at the T-tubule–sarcoplasmic reticulum junction and is currently classified among genes with at least moderate evidence for HCM, with reports of heart-failure-prone presentations in selected families [63,64,65,66].

*FHOD3* (formin-related) has been proposed as a genetic substrate for HCM in cohort- and family-based studies, consistent with its role in cytoskeletal regulation, actin dynamics, and myofibrillar architecture [31,67,68]. *ALPK3* represents a particularly instructive example, as it was historically associated with recessive disease, but more recent data support autosomal dominant inheritance in the presence of truncating variants, suggesting dose-related mechanisms and a phenotype that may vary in age at onset and severity [69].

An emerging topic in the genetics of HCM concerns so-called “elusive” variants that escape conventional genetic testing focused on coding regions and canonical exon–intron junctions. Despite the inclusion of well-validated sarcomeric genes in diagnostic panels, up to ~50% of patients with a clear clinical HCM phenotype remain genotype-negative, suggesting a genetic component not captured by traditional sequencing approaches [70,71]. A previously “hidden” proportion of patients classified as genotype-negative can be explained by variants not captured by conventional testing strategies [10,31,61].

Whole-gene locus sequencing studies and whole genome sequencing (WGS) have shown that deep intronic variants, located even hundreds of bases away from canonical splice sites, can activate non-canonical splice sites, lead to the inclusion of pseudoexons in the mature transcript, or alter regulatory elements of splicing and transcription [72,73]. Such variants are not captured by targeted panels or standard exome sequencing, but have been identified in loci such as MYBPC3, VCL, PRKAG2, and TTN in cohorts of initially “genotype-negative” HCM patients [74,75,76]. In the best-characterized cases, for example, the MYBPC3 c.1224-52G>A variant causes the inclusion of 50 intronic nucleotides in the mRNA, resulting in a frameshift and a premature stop codon, and has been observed in a significant proportion of HCM probands who were previously genotype-negative [71,77,78,79].

The use of WGS has further highlighted that up to approximately 9% of gene-elusive HCM cases can be resolved through the identification of non-coding variants that disrupt splicing or other regulatory functions not detected by targeted analyses [70]. These observations underscore not only the limitations of targeted and exome sequencing in establishing a comprehensive molecular diagnosis, but also the importance of complementary approaches such as RNA-seq or functional splicing studies to confirm the pathogenic effect of deep intronic variants [71,80,81,82]. RNA sequencing can directly reveal aberrant transcripts, including exon skipping or the inclusion of cryptic exons generated by intronic variants, thereby providing functional evidence of their pathogenicity. In parallel, emerging long-read sequencing technologies allow a more comprehensive characterization of complex genomic regions, structural variants, and full-length transcripts that may be missed by short-read approaches. In addition, growing evidence suggests that non-coding regulatory elements, variants affecting microRNAs, and promoter regions may contribute to the “missing heritability” of HCM, substantially expanding the portion of the genome that should be interrogated in patients with negative genetic testing but a typical clinical phenotype [83,84]. Taken together, these advances suggest that future diagnostic strategies for HCM may increasingly rely on integrated genomic and transcriptomic approaches, combining WGS with transcript-level analyses to improve the diagnostic yield in currently genotype-negative patients.

## 6. Phenocopies and Diagnostic Red Flags: Why “Genotype-First” Can Mislead Without Phenotype Anchoring

A clinically meaningful approach to HCM requires recognizing that increased left ventricular wall thickness may represent the final common pathway of multiple disorders (“phenocopies” or mimics) with distinct prognosis and disease-specific therapies; in adults, phenocopies account for a minority of cases (reported < 5% in some series), but their prevalence rises substantially in paediatric populations (up to ~25%), making etiological discrimination particularly critical across the life course [85].

**Figure 5:**
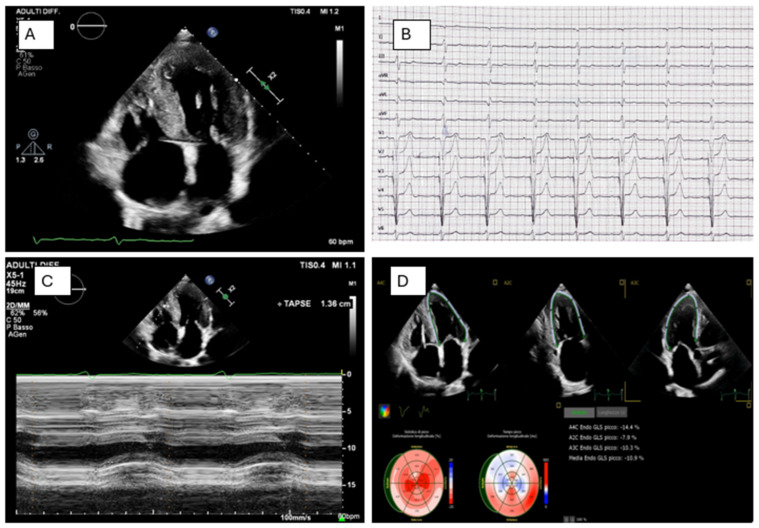
Reclassification from genotype-negative HCM to wild-type transthyretin cardiac amyloidosis (ATTRwt). The patient was initially diagnosed with genotype-negative HCM. Subsequent multimodality evaluation led to the diagnosis of wild-type transthyretin cardiac amyloidosis (ATTRwt). Panel (**A**): Apical four-chamber echocardiographic view showing a left ventricle with preserved internal dimensions and severe, diffuse, uniform wall thickening, including papillary muscle hypertrophy, with a characteristic “ground-glass” myocardial appearance. Moderate global systolic dysfunction is present (biplane Simpson LVEF 46%), mainly due to marked hypokinesia of basal segments. The left atrium is severely dilated. The interatrial septum appears thickened and hyper-reflective. The mitral valve leaflets are thickened and stiff, with chordal calcifications. Panel (**B**): Twelve-lead ECG demonstrating a ventricular paced rhythm from a pacemaker programmed in DDD mode at 50 bpm. Panel (**C**): Echocardiographic assessment of the right ventricle revealing right ventricular systolic dysfunction (TAPSE 13 mm). Panel (**D**): Myocardial deformation analysis showing a moderately reduced global longitudinal strain (GLS −10.9%), with more pronounced reduction in basal longitudinal strain values and relative apical sparing, consistent with cardiac amyloidosis.

Experts emphasize that only a limited number of inherited and acquired conditions represent true “HCM phenocopies”, characterized by increased left ventricular wall thickness mimicking sarcomeric HCM while arising from distinct molecular mechanisms and carrying specific therapeutic implications (Figure 5). These include Anderson–Fabry disease (GLA gene, X-linked), Danon disease (LAMP2 gene, X-linked), PRKAG2-related cardiomyopathy (PRKAG2 gene, autosomal dominant), and transthyretin amyloid cardiomyopathy (hereditary or wild-type ATTR) [86,87]. Additional conditions, such as RAS–MAPK pathway disorders, mitochondrial diseases, and selected glycogen storage disorders, may present with hypertrophic or HCM-like phenotypes predominantly in paediatric or syndromic contexts and should be considered in age- and phenotype-specific scenarios rather than as routine alternatives in adult HCM evaluation [85,88,89]. Age at presentation provides an important contextual discriminator, with infancy or childhood onset strongly suggesting metabolic or syndromic disease, whereas late-onset hypertrophy, particularly beyond the sixth decade, should prompt evaluation for ATTR amyloidosis.

The diagnostic challenge is that phenocopies may present with an “HCM-like” morphology while diverging in multisystem involvement, electrocardiographic signatures, and tissue phenotype; a multiparametric, phenotype-anchored diagnostic workflow (“cardiomyopathic mindset”) is required, integrating family history, extracardiac clues, electrocardiography, echocardiographic assessment including strain analysis, and cardiovascular magnetic resonance with tissue characterization, in order to guide targeted biochemical and genetic testing and to avoid misattribution of causality to incidental variants [33,90,91] (Table 2).

Practical red flags begin with inheritance and systemic features: absence of male-to-male transmission should prompt consideration of X-linked aetiologies (e.g., Fabry/Danon), whereas matrilineal patterns and neuromuscular signs suggest mitochondrial disease; extracardiac manifestations may be subtle unless actively sought (e.g., angiokeratoma, neuropathic pain, ocular/auditory involvement in Fabry; skeletal muscle weakness in PRKAG2 and mitochondrial disease; dysmorphism, lentigines, pulmonary stenosis in RASopathies) [87,92,93,94].

**Figure 6:**
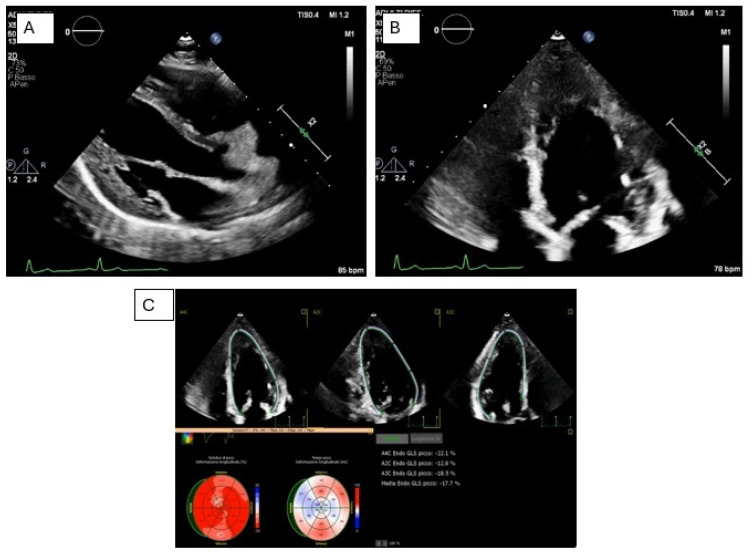
Echocardiographic findings in a young patient with Fabry disease. Panel (**A**). Parasternal long-axis view showing a left ventricle with normal internal dimensions and mildly increased wall thickness, predominantly involving the mid and apical inferior, inferolateral, and lateral segments (maximum wall thickness 14 mm; interventricular septum 9 mm; posterior wall 9 mm). Global systolic function is at the lower limit of normal (biplane Simpson LVEF 52%). No regional wall motion abnormalities are observed. Panel (**B**). Apical four-chamber view with apical zoom confirming preserved LV cavity size and mild segmental hypertrophy, particularly the apex. Panel (**C**). Myocardial deformation analysis demonstrating impaired longitudinal function with a reduced global longitudinal strain (GLS −16.9%).

ECG and imaging can provide high-yield discriminators: pre-excitation and conduction disease in the setting of marked LVH should raise suspicion for Danon and PRKAG2 cardiomyopathy (while also occurring in selected storage/mitochondrial conditions); conversely, a discrepancy between the magnitude of LVH and low QRS voltages supports cardiac amyloidosis [86,95,96,97,98]. On echocardiography, amyloidosis is classically associated with relative apical sparing, whereas Fabry disease may show concentric (sometimes biventricular) hypertrophy, disproportionate papillary muscle hypertrophy and early strain abnormalities [99,100,101,102] (Figure 6).

**Figure 7:**
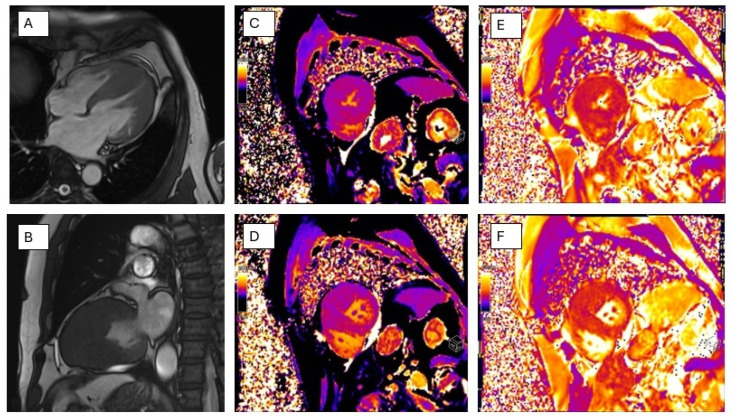
Findings in a patient with biventricular HCM. Panels (**A**,**B**) (cine CMR views): Severe asymmetric left ventricular hypertrophy with a mid-apical distribution, associated with preserved ventricular volumes and systolic function. Concomitant mid-apical right ventricular hypertrophy is observed, with preserved right ventricular volumes and systolic performance. Panels (**C**,**D**): Increased native T1 values, consistent with diffuse myocardial tissue abnormalities. Panels (**E**,**F**): Myocardial signal hyperintensity on T2-weighted images involving the mid IVS. T2 mapping demonstrates elevated T2 values at the level of the anterior wall and the interventricular septum, predominantly in the basal segments, as well as in the mid and apical inferior septum, suggestive of myocardial edema/inflammatory involvement.

CMR tissue characterization is increasingly central for phenocopy discrimination (Figure 7): mapping techniques add pathophysiological specificity beyond late gadolinium enhancement (LGE) distribution, with low native T1 values supporting sphingolipid storage in Fabry (including very early myocardial involvement that may precede overt hypertrophy), and high native T1 values supporting amyloid infiltration; these signatures can be “pseudo-normalized” in Fabry in regions with established fibrosis, reinforcing the importance of interpreting mapping and LGE together rather than in isolation [103,104,105,106,107,108].

Correct etiological diagnosis has immediate therapeutic consequences and has become more urgent in the contemporary era: Fabry disease has disease-modifying options (enzyme replacement and chaperone therapies); TTR amyloidosis has targeted pharmacological and RNA-based therapies; and, critically, the advent of myosin inhibitors increases the risk that treating an unrecognized phenocopy as “sarcomeric HCM” may be ineffective or potentially harmful—thereby making phenocopy exclusion a prerequisite for precision therapy selection [91,109,110,111,112].

## 7. Conclusions

HCM is no longer adequately described as a purely monogenic sarcomeric disorder but rather as a genetically complex condition in which rare pathogenic variants, modifier genes, polygenic background, and non-genetic factors jointly determine disease expression. While a restricted set of sarcomeric genes—most notably *MYBPC3* and *MYH7*—constitutes the core monogenic substrate of HCM and provides high diagnostic value, expanding genetic panels beyond well-validated genes increases interpretative uncertainty without proportional clinical benefit.

Incomplete and age-dependent penetrance, variable expressivity, and heterogeneous clinical trajectories—even among carriers of the same variant—limit the prognostic value of genotype alone. Differences between missense and truncating variants, dose-dependent mechanisms, and variability in protein quality control offer a biological explanation for this heterogeneity. Emerging genes and previously unrecognized variant classes, including deep intronic variants in *MYBPC3*, further expand the diagnostic landscape but require rigorous gene–disease validity assessment and phenotype integration.

Clinically, these data support a targeted and integrative approach to genetic testing, in which results inform diagnosis and family screening, while risk stratification remains primarily phenotype-driven and longitudinal. Future advances will depend on the development of integrated models combining rare variants, polygenic risk, imaging, and biomarkers to translate genetic complexity into meaningful precision care in HCM.

## Figures and Tables

**Table 1:** Gene–disease validity classification for selected genes associated with hypertrophic cardiomyopathy.

Gene	Functional Category	Level of Evidence According to ClinGen
*MYBPC3*	Sarcomeric thick filament	Definitive
*MYH7*	Sarcomeric thick filament	Definitive
*TNNT2*	Sarcomeric thin filament	Definitive
*TNNI3*	Sarcomeric thin filament	Definitive
*TPM1*	Sarcomeric thin filament	Definitive
*MYL2*	Sarcomeric thick filament	Definitive
*MYL3*	Sarcomeric thick filament	Definitive
*ACTC1*	Sarcomeric thin filament	Definitive
*ALPK3*	Kinase/sarcomere regulation	Definitive/Strong
*FHOD3*	Cytoskeletal/actin regulation	Definitive
*JPH2*	Excitation–contraction coupling	Moderate

**Table 2:** Major phenocopies of hypertrophic cardiomyopathy and key clinical features useful for differential diagnosis.

Condition	Gene Involved	Key Clinical Clues	Imaging Features	Extracardiac Features
**Fabry disease**	GLA	Adult-onset LVH, short PR interval	Concentric LVH, low native T1 on CMR	Neuropathic pain, angiokeratomas, renal dysfunction
**Danon disease**	LAMP2	Early severe hypertrophy, arrhythmias	Massive LVH, systolic dysfunction	Skeletal myopathy, cognitive impairment
**PRKAG2 syndrome**	PRKAG2	LVH with pre-excitation (WPW)	Hypertrophy often with conduction disease	Glycogen storage phenotype
**ATTR amyloidosis**	Wild type or TTR	HFpEF in elderly patients	Increased wall thickness with apical sparing on strain	Neuropathy, carpal tunnel syndrome
**Mitochondrial cardiomyopathy**	mtDNA mutations	LVH with multisystem disease	Variable hypertrophy	Diabetes, hearing loss, neuromuscular disease

## Data Availability

No new data were created or analyzed in this study.

## Abbreviations

The following abbreviations are used in this manuscript:

AF	Atrial fibrillation
ACTC1	Actin, Alpha, Cardiac Muscle 1
ALPK3	Alpha Kinase 3
ATTR	Transthyretin
ATTRwt	Transthyretin wild-type
BAG3	BCL2-Associated Athanogene 3
CMR	cardiac magnetic resonance
DCM	Dilated cardiomyopathy
DSP	desmoplakin
ESC	European Society of Cardiology
FHOD3	Formin Homology 2 Domain Containing 3
FLNC	Filamin C
GLA	Galactosidase Alpha
GLS	Global longitudinal strain
GWAS	Genome-wide association studies
HCM	Hypertrophic cardiomyopathy
HSPB7	Heat Shock Protein Family B (Small) Member 7
IVS	Interventricular septum
JPH2	Junctophilin 2
LAMP2	Lysosome-Associated Membrane Protein 2
LGE	Late gadolinium enhancement
LMNA	Lamin A/C
LP	Likely pathogenetic
LVEF	Left ventricular ejection fraction
LVH	Left ventricular hypertrophy
LVOT	Left ventricular outflow tract
MYBPC3	Myosin-Binding Protein C 3
MYH7	Myosin Heavy Chain 7
MYL2	Myosin Light Chain 2
MYL3	Myosin Light Chain 3
NGS	Next-generation sequencing
P	Pathogenetic
PLN	phosholamban
PRKAG2	Protein Kinase AMP-Activated Non-Catalytic Subunit Gamma 2
PRS	Polygenic risk scores
RBM20	RNA binding motif protein
SCD	Sudden cardiac death
SNPs	single nucleotide polymorphisms
TMEM43	Transmembrane Protein 43
TNNI3	Troponin I3
TNNT2	Troponin T2
TPM1	Tropomyosin 1
TTN	Titin
VCL	Vinculina
VUS	Variants of uncertain significance
WGS	whole genome sequencing
